# Cerebellar Liponeurocytoma Mimicking Medulloblastoma: Case Report of a Childhood and Literature Review

**DOI:** 10.3389/fonc.2021.759581

**Published:** 2021-11-25

**Authors:** Changhui Dong, Yining Jiang, Liyan Zhao, Yubo Wang, Yang Bai, Ying Sun, Yunqian Li

**Affiliations:** ^1^ Department of Neurosurgery, Central Hospital of Changchun, Changchun, China; ^2^ Department of Neurosurgery, First Hospital of Jilin University, Changchun, China; ^3^ Department of Clinical Laboratory, Second Hospital of Jilin University, Changchun, Jilin, China

**Keywords:** case report, cerebellar liponeurocytoma, children, diagnosis, liponeurocytoma, treatment, prognosis

## Abstract

**Background:**

Cerebellar liponeurocytoma is a rare benign neoplasm of the central nervous system, which arises mainly in adult patients with only 3 cases reported in children. Due to its rarity, the diagnosis and treatment strategies for cerebellar liponeurocytoma remain unclear. The purpose of this study was to explore the epidemiology, clinical features, imaging findings, pathological characteristics, different diagnoses, treatment, and prognosis of cerebellar liponeurocytoma in juveniles.

**Case Description:**

A 5-year-old boy was admitted to the department of neurosurgery due to a 5-month history of headaches, nausea, vomiting, dizziness, dysphoria, as well as visual blurring associated with the peak of the headache. Magnetic resonance imaging showed a 4.9×5.4×6.2 cm mass located in the fourth ventricle and cerebellar vermis combined with hydrocephalus and periventricular edema. The mass was completely removed, and pathological examination indicated a cerebellar liponeurocytoma of the World Health Organization Grade II classification.

**Conclusion:**

The present study was the first to report a cerebellar liponeurocytoma with total tumor resection and adjuvant radiotherapy in a pediatric patient. Total tumor resection and postoperative radiotherapy together with close and long-term follow-up seem to be the optimal treatment strategy for juvenile patients. However, the side-effect of radiation needs to be considered.

## Introduction

Cerebellar liponeurocytoma (cLNC) is a rare tumor of the central nervous system (CNS) that mostly affects the adult population and is mainly located in the posterior fossa of the brain, with the cerebellar hemisphere being the most common anatomical location, followed by the cerebellar vermis (1–3). In 1978, cLNC was first reported by Bechtel et al. as a mixed mesenchymal and neuroectodermal tumor (4). Since then, previous studies have proposed various nomenclatures like neurolipocytoma (5), lipomatous glioneurocytoma, lipidized mature neuroectodermal tumor, medullocytoma, lipidized medulloblastoma (6), and lipomatous gliomaneurocytoma (5, 7). In 2000, the World Health Organization (WHO) classification of tumors of the CNS categorized cLNC, for the first time, as a distinct entity within the neuronal and mixed neuronal-glial tumor section, named cerebellar liponeurocytoma, thereby emphasizing its neurocytic differentiation and classifying it into a group of neuronal tumors with a separate Grade I entity (8). However, longer clinical observations showed a higher likelihood of tumor recurrence rates than originally expected. Therefore, ultimately, in the WHO 2007 classification of CNS tumors, cLNCs were regarded as WHO grade II tumors (1, 9), with the same status as in the 2016 WHO classification (1, 2, 10).

When reviewing all publications involving cLNCs that were retrieved from PubMed, only 66 cases were found, and almost all of them were sporadic cases. cLNCs in children are extremely rare, with only 3 reported cases (11–13). We herein present a case of cLNC located in the 4^th^ ventricle and cerebellar vermis of a 5-year-old boy and summarized all the reported cLNCs developed in juveniles. In addition, we reviewed the available medical and scientific literature, and summarized the radiological and pathological features of this rare tumor entity, with epidemiology, diagnosis, surgery, adjuvant therapy, and prognosis being discussed in detail.

## Case Report

### History and Examination

A 5-year-old right-handed boy was admitted to the neurosurgery department with 5-month history of headache, nausea, vomiting, dizziness, and dysphoria, as well as visual blurring associated with the peak of the headache. In nearly a month, the boy’s clinical manifestations gradually worsened. He complained of being unable to maintain balance while walking or standing. The patient had no similar clinical history previously reported in his family. Moreover, neurological examination showed that the patient had bilateral grade II papilledema, gait ataxia, and was positive for the finger-nose and the Romberg test.

### Neuroimaging Findings

**Figure 1:**
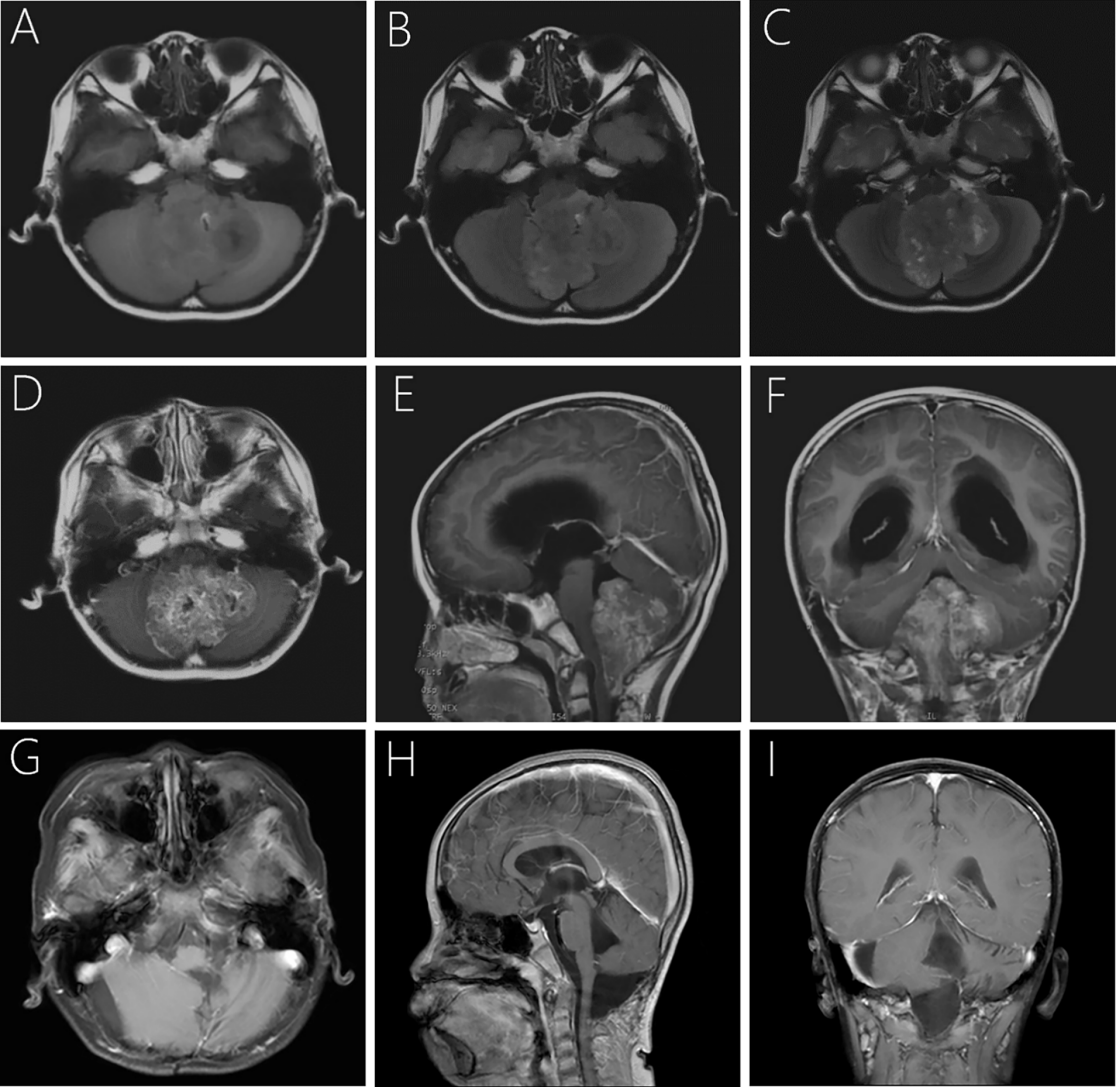
Pre- and postoperative imaging of the child. Preoperative MRI showed hypo- and isointensity mixed signal, together with focal hyperintensity on T1WI **(A)** and T2-weighted FlAIR **(B)**. The lesion is iso- and hyperintensity mixing signals and well-circumscribed on axial T2WI **(C)**. Post-contrast T1WI showed moderate heterogeneous contrast enhancement of the lesion **(D–F)**. Follow-up MRI at 29-month after surgery revealed that the lesion was completely removed with no signs of recurrence **(G–I)**.

A computerized tomography (CT) scan showed a heterogeneous dense large mass in the 4^th^ ventricle of the brain, combined with unclear edges and significant dilation of bilateral and 3^rd^ ventricles. Magnetic resonance imaging (MRI; **Figure 1**) revealed a large, irregular, heterogeneous mass of approximately 4.9×5.4×6.2 cm, which herniated to the orientation of the foramen magnum, extending to the C2 level. In addition, the bilateral cerebellar hemisphere, cerebellar tonsils, medulla oblongata, and cervical cord were particularly compressed. Iso- and hypointensity signals were seen on T1-weighted imaging (T1WI; **Figure 1A**) and T2-weighted fluid attenuated inversion recovery (FLAIR; **Figure 1B**). Mixing isointensity and hyperintensity signals were seen on T2-weighted imaging (T2WI; **Figure 1C**). On enhancement, the tumor showed moderate heterogeneous enhancement with an ill-defined boundary (**Figures 1D–F**). In addition, there was significant presence of hydrocephalus and periventricular edema.

### Surgery

A preoperative diagnosis of medulloblastoma was made and the surgery was performed under preoperative and intraoperative neuronavigation together with electrophysiological monitoring of cranial nerves VII, IX, X, XI, brainstem, somatosensory, and muscle-evoked potentials. The patient underwent midline suboccipital craniectomy, C1 posterior arch excision, and total tumor resection in a lateral prone posture. The tumor appeared pinkish, solid, and bloody. The interface between the tumor and healthy parenchyma was not as sharply defined.

### Pathological Findings

**Figure 2:**
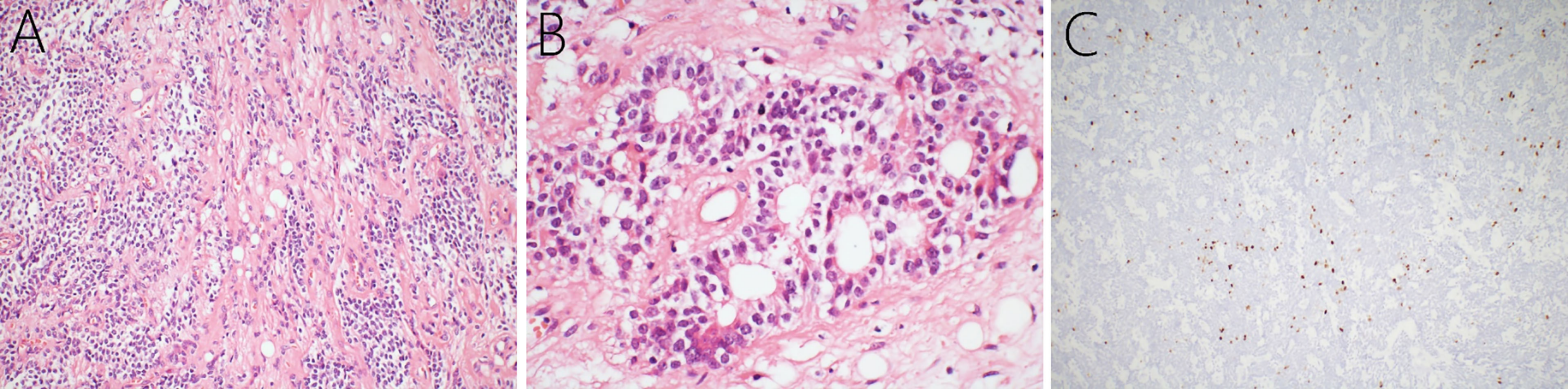
Histopathology of the cerebellar liponeurocytoma. Hematoxylin and eosin-staining showing that a large number of small round tumor cells with low mitotic activity grow in sheets, and clusters of fat cells **(A)** and that chrysanthemum clusters **(B)** are seen among the cells. Immunohistochemical examination presented a Ki-67 index of 5% grossly, and 10-20% focally **(C)**.

After combined diagnosis of two chief pathologists at our hospital and the Xuanwu Hospital Capital Medical University, China, the pathological diagnosis was cLNC, with WHO Grade II. Upon histopathological examination, hematoxylin and eosin-stained paraffin sections showed predominantly small to moderately, round or ovoid tumor cells, with low mitotic activity. These tumor cells grew in a sheet shape with clusters of fat cells were observed in the center (**Figure 2A**) or formed chrysanthemum-liking clusters (**Figure 2B**). Immunohistochemical analysis indicated that the Ki-67 index was ± 5% mostly, and only 10-20% focally (**Figure 2C**). In addition, the neoplasm was positive for synaptophysin, glial fibrillary acidic protein (GFAP), neuron-specific enolase (NSE), microtubule associated protein-2 (MAP-2), vimentin, and negative for isocitrate dehydrogenase-1 (IDH1)-R132H, neurofilament, oligo-2, S-100, and epithelial membrane antigen (EMA).

### Postoperative Course

The patient’s postoperative course was uneventful, and he discharged on the 14^th^ day with ataxia and the symptoms of pontine reticular formation lateral-gaze center injury. The patient received postoperative radiotherapy with a dose of 5400cGray/25F in the tumor bed and 4500cGray/25F in a high-risk area, respectively. We then processed a strict and long-term follow-up for 29-months until now. Postoperative MRI of brain and the whole spinal cord together with the periodical cranial MRI examinations demonstrated that the lesion had been completely removed, with no signs of recurrence or metastasis (**Figures 1G–I**). The symptoms of ataxia and binoculus gazing to the right showed some improvement 29 months postoperatively, and we will continue the follow-up the patient closely. The child and his parents signed consent to take part in the study.

## Discussion

### Epidemiology

cLNCs are highly uncommon, benign, slow-growing glioneuronal neoplasms of the CNS with a favorable clinical prognosis, but a high likelihood of recurrence (1, 9), which develop mainly in the adult population. The cerebellar hemisphere is the most common anatomical location for these tumors, followed by the cerebellar vermis, and the tumor can also occur in the supratentorial parenchyma (10, 11, 14–16). Anghileri et al. also reported a lumbar metastasis 11 years after the primary surgical excision (14). Based on these studies, some researchers had given advice to replace the term ‘cerebellar liponeurocytoma’ into ‘central liponeurocytoma’, or solely as ‘liponeurocytoma’ (16–18). In all four pediatric patients with liponeurocytoma, including the case reported here, the onset location included the 4^th^ ventricle and cerebellar vermis (2/4) (12), left cerebellar hemisphere (1/4) (13), and right frontal lobe (1/4) (11). The onset age of juvenile patients in two females and two males was 4-11 years, with a mean age of 6.75 years. Due to its rarity, little is known about the underlying causes of cLNCs. The cause and origin of the neoplasm have been suggested as a familial predisposition with possible autosomal dominant mode of inheritance in recent reports (19).

### Clinical Presentation

The clinical presentation in children is not specific and depends on the tumor’s location and mass effect (**Table 1**). Typically, pediatric patients may present with symptoms of dizziness, unsteadiness, gait disturbance, frequent falls, and symptoms of high intracranial pressure, including headaches, vomiting, nausea, and papilledema (3). Obstructive hydrocephalus and flow obstruction of the cerebrospinal fluid may be present (3). On neurological examination, patients usually show signs of ataxia, gait disturbance, and focal neurologic deficits, especially in the posterior cranial nerves. Headaches complicated with nausea and vomiting were observed in all four children. Moreover, in pediatric patients, the tumor volume is generally large, which might associate with the compensation of unclosed or not firmly integrated cranial sutures of children. Furthermore, the average onset time was 2.13 months (ranged from 2 weeks to 5 months), which was significantly shortened than that when compared with the adult patients (10.12 months).

**Table 1:** The clinical characteristics of four pediatric patients with liponeurocytoma.

Author^reference^/ year	Age(y)/ sex	Onset of symptoms	Clinical presentation	Location	CT	MRI	Tumor volume	Hydrocephalus	Peritumoral edema	Preoperative diagnosis	Sugary	Postoperative treatment	Recurrence	Reoperation	Follow-up
Present case	5/M	5 months	Headache, nausea, vomiting, dizziness, and dysphoria, as well as visual blurring associated with the peak of the headache.	The 4^th^ ventricle and cerebellar vermis	A heterogeneous dense large mass in the 4^th^ ventricle, combined with unclear edges and significant dilation of bilateral and 3rd ventricles.	Iso- and hypo- signals on T1WI and FLAIR. Mixing iso- and hyper- signals on T2WI. Mild to moderate heterogeneous enhancement.	4.9×5.4×6.2 cm	Y	Y, mild	Medulloblastoma	GTR	Y, postoperative radiotherapy of 5400cGray/ 25F	None	None	29-month follow-up without tumor residue or recurrence.
Cai (11)/ 2018	11/M	2 months	Intermittent headache with nausea and vomiting in the morning.	Right frontal lobe	An irregular hypodense and iso- mixed mass.	Cystic-solid mass, the solid part exhibited heterogeneous iso- and several hypo- spots on T1WI and T2WI; the cystic part showed uniform hypo- on T1WI and hyper- on T2WI. Solid part and cystic wall were clearly enhanced.	4.5×4.7×6.4 cm	N	Y, moderate	Oligodendroglioma	GTR	None	None	None	74-month follow-up without recurrence.
Nzegwu (13)/ 2016	6/F	4 weeks	Headache and vomiting for 4 weeks. Headache was insidious, dull, predominantly left sided, worse in the morning associated with vomiting which relieves it. Gait disturbance.	Left cerebellar hemisphere	Left cerebellar tumor with obstructive hydrocephalus.	Iso- and hypo- heterogeneous signals on T1WI; hyper- signal on T2WI. (No other detailed information available).	N/A	Y	Y, mild	N/A	GTR	None	None	None	Radiotherapy is possible if the condition not stable. (No detailed information available).
Jouvet (12)/ 2005	4/F	2 weeks	Intracranial hypertension with headache, associated with nausea and vomiting.	The 4^th^ ventricle	N/A	Hypo- signal on T1WI and hyper- on T2WI; heterogeneous enhanced mass was seen after enhancement. Relapse: two homogeneous nodules comprised in a mass, with hypo- on T1WI and iso- on T2WI. Homogeneous nodule enhancement showed on postcontrast MRI.	3×2×5 cm; Relapse: two masses of 3×1.5×2 cm and 1.2×8 cm, respectively.	Y	N/A	Medulloblastoma	ITR	None	14 months postoperatively.	Y	The tumor relapsed and performed a second GTR 14-month after the first ITR (no detailed information available).

N/A, not available.

### Radiologic Characteristics

On CT scans, cLNCs usually present as well-demarcated and heterogeneous hypodense or isodense solid masses, with or without the presence of microcysts in the tumor (11, 20, 21). Analysis of MRI scans shows that these tumors are usually heterogeneous, well-circumscribed with lipid content (22). cLNCs always present hypointensity or isointensity on T1WI and hyperintensity on T2WI (3, 19). Focal hyperintensity on T1WI and T2WI, and hypodensity on CT scans from the tumor are characteristic findings, which remind fatty tissue, and may help determine cLNCs preoperatively (3, 11). In addition, hyperintensity can be observed on FLAIR imaging and diffusion-weighted imaging (3, 7). Enhanced MRI shows heterogeneous contrast enhancement in most cLNCs (3, 7, 10, 19). In general, the imaging features of juvenile patients are consistent with that of adults. Notably, mild to moderate peritumoral edema was observed in three pediatric patients (3/4), with two cases of mild, and one case of moderate surrounding edema. However, peritumoral edema was observed only in 9 adult patients (9/63, [14.3%]) (16, 23–30), as was mild edema. In some studies, the metabolic activity of cLNCs was evaluated with positron emission tomography (PET) (7, 31). In [18F] fluorodeoxyglucose PET, lower cLNC tumor accumulation was observed, when compared to the normal cerebellar cortex, showing a lesion-to normal cerebral cortex accumulation ratio of 0.62. On [11C] methionine PET, the cLNC tumor showed higher accumulation, with a lesion-to-normal cerebral cortex accumulation ratio of 2.73.

### Pathological Features

Pathological examination is the most important tool for diagnosing cLNCs. We summarized the pathological characteristics of the four pediatric cases studied in **Table 2** and found the histopathological features for juveniles to be consistent as in adults. cLNCs consistently show lipidized cells arranged in clusters or scattered between small tumor cells with ill-defined borders (3). The tumor cells can be round, spindle-shaped (15) or pleomorphic with clear eosinophilic cytoplasm (2, 3, 10, 11). Moreover, mitosis is rare or absent in these cells. Notably, although most cLNCs show benign histological features, several atypical features of cLNCs have been documented, including hyperchromatic nuclei, nuclear and cytoplasmic atypia, increased mitoses, focal necrosis, microvascular proliferation, prominent astrocytic components, and a high proliferation index (12, 16, 32). However, in the new 2016 WHO classification of brain tumors, there is no consensus regarding atypical cLNCs (1).

**Table 2:** Pathological features of four pediatric patients with liponeurocytoma.

Author^reference^/ year	Gross inspection	Hematoxylin and eosin staining	Immunohistochemistry analysis
Present case	Pinkish, solid and bloody (intraoperative findings).	Predominantly small to moderately, round or ovoid tumor cells, with slow mitotic activity. Lipidized cells were found arranged in clusters and scattered between small tumor cells.	SYN(+), NSE(+), GFAP(+), MAP-2(+), Vimentin(+); IDH1-R132H(-), NF(-),oligo-2(-), S-100(-), EMA(-); Ki-67 index was ± 5% mostly, and 10-20% focally.
Cai (11)/ 2018	Gray-white, cystic-solid, filled with yellowish fluid (intraoperative findings). Gross specimen: grayish appearance with lipoid tissue.	Isomorphic, small, round neoplastic cells resemble neurocytes and focal lipomatous differentiation. The tumor cells showed round nuclei, clear cytoplasm, and close arrangement.	SYN(+), MAP-2(+), NeuN(+), GFAP(+); Olig-2(-); MIB-1 antibody immunolabeling approximately 1.5%.
Nzegwu (13)/ 2016	Soft, grey-yellow mass with partly cystic consistency; a fairly well-defined brain-tumor boundary (intraoperative findings).	An extensively lipidized tumor composed of sheets of mature adipose tissue with some neurocytic cells in the background minority with hyalinized blood vessels. No mitosis was seen.	S-100(+); desmin(+).
Jouvet (12)/ 2005	N/A	Initial: Uniform round small cells with prominent areas of lipidization and vascular septa. The cells had round nuclei and small nucleoli, finely specked chromatin, and a scant or oligo-like cytoplasm. Lipidized cells were arranged in groups or scattered throughout the tumor tissue. The tumor cells sometimes had a signet ring appearance. No fibrillary background or rosettes were identified. Necrosis and microvascular proliferation were absent and mitoses were rare. Recurrence: Some regions were histologically identical to the original tumor, but with fewer adipose-like cells, while others presented an endocrine architecture with oligolike or pleiomorphic cells. The tumor cells contained round to oval or irregular nuclei and were sometimes bi- or multinucleate and contained varying quantities of cytoplasm and vacuoles. Rosette-like arrangements of tumor cells around the thin vessels were observed in other areas, which had features similar to cellular ependymoma.	Initial: Strong positive: SYN, Vimentin, EMA, KP1; Moderate positive: Chromogranin A; Weak or variable: NF, GFAP, S-100; Absent/ very low: Cytokeratin; Ki-67 index about 10-15%. Recurrence: Strong positive: SYN, Chromogranin A, Vimentin, S-100, EMA, Cytokeratin, KP1; Moderate positive: GFAP; Weak or variable: NF; Ki-67 index about 15-30%.

SYN, synaptophysin; NSE, neuron-specific enolase; GFAP, glial fibrillary acidic protein; MAP-2, microtubule associated protein-2; IDH-1, isocitrate dehydrogenase-1; NF, neurofilament; EMA, epithelial membrane antigen; N/A, not available..

Ki-67 is a nuclear protein that is associated with cellular proliferation (1). Higher Ki-67 levels usually associate with a higher risk of recurrence (17). In pediatric patients, a high Ki-67 proliferation index was observed in two children (Jouvet and the present case) (12). In the case of Jouvet et al., a Ki-67 index of 10-15% was observed at the time of initial surgery, and 15-30% was observed in recurrent cLNC (12). In our case, Ki-67 expression was grossly 5%, and 10-20% focally. Gembruch et al. performed a systematic review for LNC in 2018 and presented that the mean value of the Ki-67 proliferation index was 3.7 ± 4.0%, and 9.2 ± 7.8% for the first tumor recurrence (3). However, characteristics of the proliferation index for pediatric patients have not been identified due to the limited number of cases. Some studies presented that the Ki-67 index had a tendency to increase after cLNCs recurrence, suggesting tumor progression with a tendency to malignant transformation (3, 12, 16). Immunostaining provides additional meaningful information that allows for correct diagnosis and differential diagnosis. cLNCs immunohistochemical analyses were positive for SYN, GFAP, NSE, and MAP-2, and negative for IDH-1 mutations, oligo-2, and EMA (10, 11, 23, 31). In addition, enhanced levels of the adipocyte-associated transcription factor, NEUROG1, and fatty acid-binding protein 4, have been described in cLNCs (14).

### Differential Diagnosis

The differential diagnosis includes medulloblastoma, oligodendroglioma, ependymoma, and central neurocytoma among others, especially for pediatric patients (7, 32–35). Medulloblastoma is one of the most important differential diagnoses for juveniles with cLNC, especially tumor located at the cerebellar vermis and the 4^th^ ventricle, which was preoperatively diagnosed in two cases of pediatric patients. Medulloblastoma always shows foamy histiocytes, primitive neuroectodermal cells, a high mitotic rate, and a high Ki-67 index (9, 34, 36). Moreover, isochromosome 17q, a genetic hallmark that is present in 40% of classic medulloblastomas, has never been observed in any of the cLNCs (36). Medulloblastomas can be genetically excluded when tested for PTCH, APC, or β-catenin mutations. These are associated with a subset of medulloblastomas but are all absent in cLNCs (36). Furthermore, cLNCs usually reveal tumor protein 53 missense mutation with a higher frequency than that in medulloblastoma (27, 37). Tumor protein 53 mutations are also absent in neurocytomas (11, 17), which help the differential diagnosis. Oligodendroglioma is another significant differential diagnosis, which was preoperatively diagnosed in one case of juvenile cLNC. Oligodendrogliomas lack immunohistochemical expression of neuronal markers, MAP-2 and SYN (20). Genetically, oligodendrogliomas are known to bear a high frequency of IDH1-R132H mutations as well as a co-deletion of chromosome 1p/19q, which were absent in cLNCs (38, 39). Furthermore, oligodendrogliomas have not shown lipomatous differentiation (11).

### Treatment

There is no golden standard for treatment guidelines for pediatric patients with cLNCs for such small cases, considering the side-effects of radiation and the quality of life. For pediatric patients who underwent gross tumor resection (GTR), if the pathological examination presented a low Ki-67 index, and no atypical histopathological findings were observed, such as necrosis, high mitotic activity, and prominent vascular hyperplasia, periodical MRI surveillance for possible recurrence was among the primary treatment principle, and radiotherapy could be avoided (2, 3, 16, 29). In the case reported by Cai et al., a cLNC in the right frontal lobe was completely resected and no recurrence was observed at 6 years and 2 months after the initial total tumor resection (11). This was also true for the case reported by Nzegwu et al. who also only received GTR (13). Regarding patients with incomplete tumor resection (ITR) and/or a high Ki-67 index, postoperative radiotherapy is strongly recommended (23). Jouvet et al. reported a cLNC with only ITR (Ki-67 index of 10-15%), and the tumor relapsed 14-month later (Ki-67 index of 15-30%) (12). Our case presented here, represents a cLNC treated by GTR and postoperative radiotherapy due to its focally high Ki-67 index of 10-20%, and this is the first reported pediatric case with cLNC who accepted surgery combined with follow-up radiotherapy, with no tumor recurrence observed in 29-months postoperatively. Similarly, no tumor recurrence was reported in adult patients who underwent GTR and adjuvant radiotherapy (3). Gembruch et al. censused all reported liponeurocytomas and suggested that recurrence was observed in 26.1% (6/23) patients for GTR without adjuvant radiotherapy; 16.7% (1/6) for patients with ITR and radiotherapy; and 77.8% (7/9) for patients with ITR but without radiotherapy (3). Regarding the radiotherapy dose, the boy in our case received a dose of 5400cGray in the tumor bed, which was in line with the radiation dose of most liponeurocytomas (3). For patients with only slightly more aggressive signs on histology, avoidance of radiotherapy is recommended (29). Notably, in either event, strict and long-term follow-up, together with periodical MRI scans are extremely important and imperative in nature. Furthermore, more reported cases and long-term follow-up studies are needed to confirm the choice of radiotherapy for pediatric patients. As for the treatment of recurrent cLNCs, there is no consensus on what the ‘optimal’ treatment should be, and the treatment protocol greatly depends on the patient’s condition (20). Jouvet et al. performed a re-operation with GTR, and no tumor recurrence was observed (12). Jenkinson et al. advocated for adjuvant radiotherapy in the case of recurrence (15).

### Prognosis

Given that there are only four known cases, the prognosis of children with cLNC is difficult to assess. Although the cLNCs were classified as WHO Grade II tumors, recurrence rates are extensively reported in adult cases, which is the reason for the WHO grade classification upgrade in 2007. In pediatric cases, only one case of recurrence was reported by Jouvet et al. with the main reason of ITR (12). ITR and atypia histological features, including a high Ki-67 index, are the main reasons for high recurrent rates (15). Therefore, total tumor resection should be the surgical aim. However, a remarkable fact is that, even when treated with total resection, recurrence may be unavoidable (14). Thus, due to the potentially long-term malignancy, close follow-up is recommended and essentially necessary.

## Conclusion

In summary, cLNC is rarely reported in the literature and, with only 3 known cases, is extremely rare in pediatric patients. Thus, more cases describing cLNC and long-term follow-up studies are warranted to fully understand cLNC in the juvenile population. Therefore, because of these limitations, our present case report might represent an additional reference among the few available that might serve as a potential guide for clinicians and clinical studies.

## Data Availability Statement

The original contributions presented in the study are included in the article/supplementary material. Further inquiries can be directed to the corresponding author.

## Ethics Statement

The studies involving human participants were reviewed and approved by Ethics Committee of the First hospital of Jilin University. Written informed consent to participate in this study was provided by the participants’ legal guardian/next of kin for the publication of any potentially identifiable images or data included in this article.

## Author Contributions

CD, YJ and LZ made study design, data collection, data analysis and interpretation, and composed the manuscript and literature review. YL, CD and YW were the surgeon that performed the surgery and did data collection, data analysis, and interpretation. YS and YB made English and grammar corrections, critical revisions, and approved final version. YL had the acquisition, analysis or interpretation of data for the work, revising it critically for important intellectual content, final approval of the version to be published, and agreement to be accountable for all aspects of the work in ensuring that questions related to the accuracy or integrity of any part of the work are appropriately investigated and resolved. All authors agree to be accountable for the content of the work. All authors contributed to the article and approved the submitted version.

## Conflict of Interest

The authors declare that the research was conducted in the absence of any commercial or financial relationships that could be construed as a potential conflict of interest.

## Publisher’s Note

All claims expressed in this article are solely those of the authors and do not necessarily represent those of their affiliated organizations, or those of the publisher, the editors and the reviewers. Any product that may be evaluated in this article, or claim that may be made by its manufacturer, is not guaranteed or endorsed by the publisher.

## Abbreviations

cLNC, cerebellar liponeurocytoma; CNS, central nervous system; CT, computerized tomography; EMA, epithelial membrane antigen; FLAIR, fluid attenuated inversion recovery; GTR, gross tumor resection; GFAP, glial fibrillary acidic protein; IDH-1, isocitrate dehydrogenase-1; ITR, incomplete tumor resection; MRI, magnetic resonance imaging; MAP-2, microtubule associated protein-2; NSE, neuron-specific enolase; PET, positron emission tomography; T1WI, T1-weighted imaging; T2WI, T2-weighted imaging; WHO, World Health Organization.
